# A Method for Efficient Calculation of Diffusion and Reactions of Lipophilic Compounds in Complex Cell Geometry

**DOI:** 10.1371/journal.pone.0023128

**Published:** 2011-08-31

**Authors:** Kristian Dreij, Qasim Ali Chaudhry, Bengt Jernström, Ralf Morgenstern, Michael Hanke

**Affiliations:** 1 Institute of Environmental Medicine, Karolinska Institutet, Stockholm, Sweden; 2 School of Computer Science and Communication, Royal Institute of Technology, Stockholm, Sweden; German Cancer Research Center, Germany

## Abstract

A general description of effects of toxic compounds in mammalian cells is facing several problems. Firstly, most toxic compounds are hydrophobic and partition phenomena strongly influence their behaviour. Secondly, cells display considerable heterogeneity regarding the presence, activity and distribution of enzymes participating in the metabolism of foreign compounds i.e. bioactivation/biotransformation. Thirdly, cellular architecture varies greatly. Taken together, complexity at several levels has to be addressed to arrive at efficient *in silico* modelling based on physicochemical properties, metabolic preferences and cell characteristics. In order to understand the cellular behaviour of toxic foreign compounds we have developed a mathematical model that addresses these issues. In order to make the system numerically treatable, methods motivated by homogenization techniques have been applied. These tools reduce the complexity of mathematical models of cell dynamics considerably thus allowing to solve efficiently the partial differential equations in the model numerically on a personal computer. Compared to a compartment model with well-stirred compartments, our model affords a more realistic representation. Numerical results concerning metabolism and chemical solvolysis of a polycyclic aromatic hydrocarbon carcinogen show good agreement with results from measurements in V79 cell culture. The model can easily be extended and refined to include more reactants, and/or more complex reaction chains, enzyme distribution etc, and is therefore suitable for modelling cellular metabolism involving membrane partitioning also at higher levels of complexity.

## Introduction

Modeling the intracellular dynamics of diffusion and reaction and its role in cellular processes such as metabolism or cellular signaling is an important aspect of systems biology [1], [2]. Using quantitative mathematical models and computer simulation the spatiotemporal behavior of chemicals, which are difficult to measure in individual cells and their organelles, can be precisely analyzed. Although lipophilic substances, both exogenous and endogenous, are of primary importance in cell toxicity, cellular signaling and behavior, the intracellular dynamics of lipophilic compounds governed by interactions with membrane lipids and partitioning phenomena have not been extensively studied. Examples of important lipophilic molecules, which are absorbed and distributed through cellular membranes to a significant degree, are lipid signaling molecules (e.g. sphingolipids [3]), vitamins (e.g. a-tocopherol [4]), drugs (e.g. cannabinoids [5]), steroids (e.g. glucocorticoids [6]), and environmental pollutants (e.g. polycyclic aromatic hydrocarbons [7]).

An intriguing challenge in developing a diffusion-reaction model including the cellular membranes is the enormous complexity of intracellular structure. A human cell consists schematically of an outer cellular membrane, a cytoplasm containing a large number of organelles (mitochondria, endoplasmic reticulum etc.), a nuclear membrane and the nucleus containing DNA. The organelle membranes create a complex and dense system of membranes or subdomains throughout the cytoplasm. Since the spatial distribution of chemicals (and their metabolites) has to be taken into account, the mathematical description leads to a system of reaction-diffusion equations in a complex geometrical domain, dominated by thin membranous structures. If these structures are treated as separate subdomains, any model becomes computationally very expensive. Previously this problem has been circumvented by using compartment models assuming fast equilibration (well-stirred compartments). Here we show that this assumption is not always valid.

In order to make our explicit cell representation numerically treatable an approach using techniques for mathematical periodic homogenization [8]–[10] and Monte-Carlo approaches as used, e.g., in groundwater transport in fractured rocks [11]–[13] was developed [14], [15]. This allowed for a manageable system of reaction-diffusion equations for the various molecular species while at the same time retaining the essential features of the metabolism under consideration. The present work is the first model describing the diffusion and reactions of lipophilic compounds using this approach.

To validate the specific model and mathematical approach the model was compared to data from *in vitro* and cell culture experiments describing the partitioning, intracellular metabolism, and reactivity of polycyclic aromatic hydrocarbons (PAHs). The PAHs are a group of highly lipophilic widespread carcinogenic environmental pollutants frequently used as model compounds in modeling different aspects of environmental pollution and toxicity [16], [17]. The results showed that lipophilicity and membrane partitioning are important parameters in the metabolism and DNA-adduct formation of these compounds. Furthermore the model and cellular experiments displayed good qualitative and quantitative agreement in describing the cellular uptake, diffusion and reactions.

## Methods

By developing an averaged model of the cytoplasm a computationally tractable model of a cell and its surrounding media can be made. In the following example the benefits of the proposed procedure for deriving effective diffusivities, reaction rates etc. is demonstrated.

**Figure 1:**
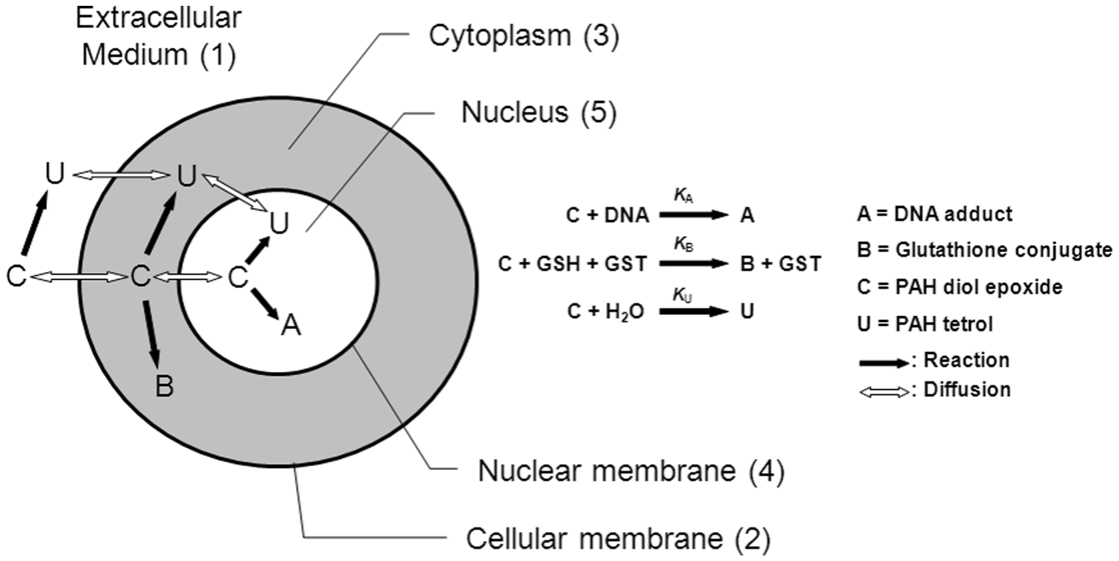
Schematic diagram showing the reactions and diffusion in and around one cell. Note that there are no reactions in the lipophilic part of the cytoplasm. Digits represent the numbering of the different subdomains.

The model describes the uptake and intracellular dynamics of the ultimate carcinogenic PAH metabolite, diol epoxides (DEs), used in our previous in vitro and cellular experiments [18]–[22]. The computational domain consists of the subdomains nucleus, nuclear membrane, cytoplasm, cellular membrane, and extracellular medium. The precise geometry used for the numerical experiments will be defined later. The transport and reactions are simplified as sketched in Figure 1. The DEs are referred to as 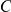. In the model no reactions take place in the membranes, or so-called lipid compartment of the cell, but only in the aqueous compartment. Inside the cytoplasm the DEs undergo two main reactions. Firstly, glutathione (GSH) conjugation, catalyzed by the enzyme family of glutathione transferases (GSTs), giving rise to DE-GSH conjugates [23], [24], referred to as 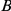 in the model. Secondly, the DEs undergo hydrolysis (reaction with water) to yield tetrols [25], [26], referred to as 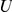. The enzymatic reaction only takes place in the aqueous part of the cytoplasm whereas hydrolysis takes place in all aqueous compartments (including the extracellular medium). Both reactions result in the elimination of the harmful DEs. The DEs will also diffuse into the nucleus and react covalently with DNA forming DNA-adducts, referred to as 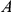. In the case of missing or erroneous DNA repair adducts/damage may result in mutations and eventually tumor development [25], [27], [28]. The concentrations of water, GST/GSH, and DNA are assumed to be constant in their respective subdomains leading to simple linear dynamics for the reactions.

Because of the lipophilic nature of the modeled compound and its metabolites a major part of the molecules will be absorbed into the cellular membranes. The partition coefficient, 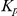, is the equilibrium ratio of the concentration of 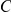 or 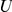 between any aqueous compartment and its adjacent lipid compartment [29]. 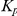 for DEs and their tetrols vary in the range 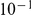 to 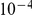. The exact values used in this experiment and for this model, as well as the values and units of all parameters in the experiment and its model can be found in Tables 1 and 2.

**Table 1:** Chemical constants for the model.

symbol	constant	value	ref.
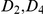	Diffusion coefficient in cell/nuclear membrane [m 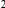 s 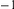 ]	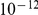	
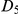	Diffusion coefficient in nucleus[m 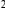 s 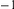 ]	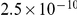	a
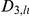	Diffusion coefficient in cytoplasm membranes/tangential[m 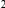 s 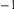 ]	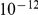	
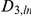	Diffusion coefficient in cytoplasm membranes/normal[m 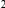 s 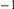 ]	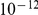	[60]
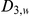	Diffusion coefficient in cytosol[m 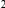 s 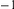 ]	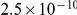	a
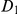	Diffusion coefficient in extracellular medium [m 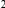 s 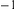 ]	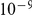	[56], [57]
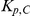	Partition coefficient for BPDE	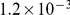	b
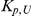	Partition coefficient for BPT	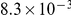	b
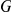	Concentration of GST [M]	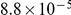	[20]
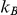 c	Catalytic efficiency [M 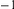 s 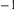 ]	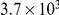	[19]
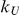	Solvolytic reactivity forming U [s 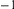 ]	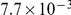	[18]
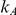	DNA adduct formation rate [s 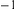 ]	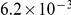	d
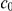	Initial concentration in extracellular medium [M]	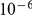	

a based on 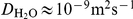 of benzo[a]pyrene [56], [57] and the relationship that 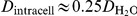 [58], [59].

b Determined using ALOGPS 2.1 software [61], [62].

c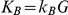.

d Not published.

**Table 2:** Geometric constants for the model.

constant	value	ref
Volume of one cell [m 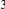 ]	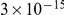	[20]
Relative thickness of cell/nuclear membrane	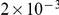	a
Volume of cell/volume of nucleus	4	
Volume of cell medium [m 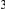 ]	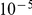	[20]
Membrane volume fraction in cytoplasm [%]	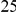	
Number of cells	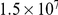	[20]

a Based on the size of V79 cells (4–8 mm) [63] and the typical cellular membrane (7–10 nm).

In this paper the following modelling assumptions are made:

**A1** We adopt the continuum hypothesis, i.e., we assume that the set of molecules in the cell can be modelled by considering a continuous representation (a concentration).

**A2** The physical and chemical properties of the cytoplasm and of the membranes are uniform.

**A3** On a small scale in space, the volume between the outer cellular membrane and the nuclear membrane consists of layered structures cytoplasm/membranes.

**A4** In a larger scale, this volume contains an unordered set of the small-scale substructures which are uniformly distributed over the volume.

**A5** Absorption and desorption is in rapid equilibrium at the membrane/cytoplasm boundary and therefore the relative concentration at the border can be conveniently described by the partition coefficient.

### Governing Equations

In the following section the mathematical model is described. Invoking assumption **A1**, the distribution of the substances is described using concentrations. With a slight abuse of notation, the concentration of a substance will be denoted by the same letter, e.g., the concentration of 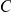 is denoted by 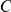 again. Moreover, in order to distinguish between the concentrations within the different compartments an index is added. For example, the concentration of 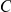 in the extracellular water (compartment 1) is given by 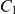. In the cytoplasm, concentrations in the aqueous and lipid parts needs to be distinguished. This will be done by using indices 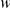 and 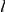, respectively. As an example, 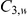 denotes the concentration of 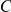 in the aqueous part of the cytoplasm. The diffusion coefficient will be denoted by 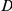 using an index corresponding to the compartment.

In the following, the gradient operator will be denoted by 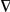. In Cartesian coordinates we have 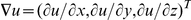. The normal derivative of a fucntion 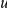 will be denoted by 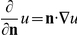.

#### Partial Differential Equations

The reaction mechanism of Figure 1 gives rise to the following system of reaction-diffusion partial differential equations.

- Subdomain 1 (extracellular medium)
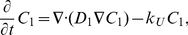
(1)

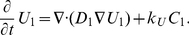
(2)
- Subdomains 2 and 4 (cellular and nuclear membranes)

For 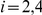, it holds:
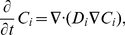
(3)

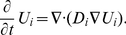
(4)

- Subdomain 5 (nucleus)
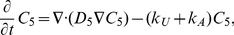
(5)

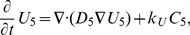
(6)

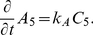
(7)
- Subdomain 3 (cytoplasm)

The cytoplasm consists of two parts, namely, the lipid (membranes) and the aqueous (cytosol) ones. The reactions take place in the aqueous part, only. This gives rise to the following equations:
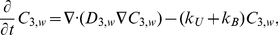
(8)

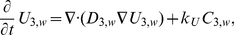
(9)

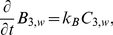
(10)

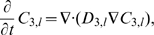
(11)

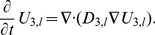
(12)


Even though, in cells, 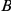 diffuses in the cytoplasm and subsequently is pumped out of the cell by multidrug resistance proteins [30], [31], the diffusion or removal of the substance 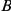 is not included. Since we are currently only interested in the total content of 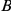 produced by the cell and since diffusion does not change the mass balance, this approach is sufficient for our purposes.

In the cytoplasmic membranes, we will distinguish between the diffusion rates normal and tangential to the membrane. So 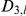 will be a tensor in contrast to all other diffusion constants which are scalar values.

As a consequence of assumption **A2**, the diffusion coefficients will be constant in their respective subdomains.

#### Transmission Conditions

The topology used for the computational domain is sketched in Figure 1. At the interfaces between subdomains 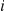 and 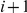, transmission conditions for 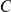 and 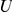 are needed. Mass conservation leads to continuity of fluxes between the different subdomains. At the interfaces between aqueous and lipid compartments, the jump of the concentrations is described by the partition coefficient 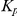,
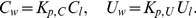
(13)The use of partition coefficients is justified because of assumption **A5**. Invoking **A2**, 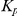 is assumed to be a constant independent of the interfaces. Note that similar transmission conditions hold true on all aqueous/lipid interfaces, for example, in the cytoplasm.

The transmission conditions at the interfaces of subdomains 1/2 and subdomains 4/5 become
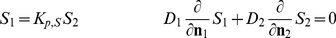
(14)

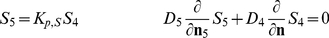
(15)for the substances 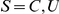. Here, 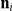 denotes the outer normal vector of subdomain 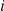. Obviously, 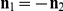 and 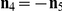.

Substance 
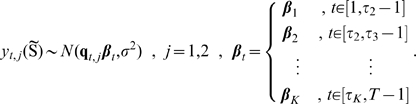
, being the covalent binding of C to the DNA, is restricted to subdomain 5 and thus not allowed to move into the other subdomains. Hence, the boundary conditions become
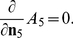
(16)Since 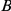 is subject to an ordinary differential equation, only, no boundary conditions for 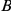 are necessary.

The description of the transmission conditions at the boundaries of the cytoplasm is slightly more complex since it consists both of aqueous and lipid parts. Let 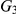 denote the subdomain occupied by the cytoplasm, 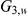 be the aqueous part, and 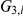 be the lipid part. For the interfaces of the cytoplasm with the compartments 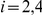 and the substances 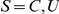 it holds:

- If 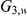 and subdomain 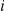 have a common interface:
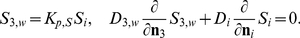
(17)
- If 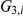 and subdomain 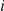 have a common interface:
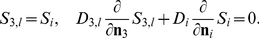
(18)

The transmission conditions between the aqueous and the lipid parts of the cytoplasm are as follows:
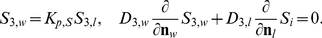
(19)


#### Boundary and Initial Conditions

For definiteness, the subdomain consisting of the extracellular water is restricted to a (sufficiently large) bounded domain. We assume that the system is closed. Hence, on the outer boundary, Neumann boundary conditions are required,
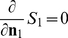
(20)for 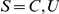. At the initial point in time it is assumed that none of the substances 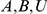 are present in the system. 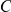 is added to the system at initial time. This gives rise to the condition
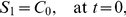
(21)with a constant 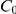 while all other concentrations are set to zero. Note that it is easy to consider the more realistic case of 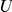 having a nonzero initial concentration in the extracellular domain due to rapid hydrolysis.

### Derivation of Effective Equations for the Cytoplasm

The geometry of the cytoplasm is very complex, containing a large number of organelles forming a complex and dense system of membranes or subdomains throughout the cytoplasm. If we would discretize Eqs. 8–12 immediately, a very fine grid would be required in order to resolve the small geometric structures. This is practically impossible given the limits of computational power. Therefore, we will derive *effective* equations for the substances in the cytoplasm. This way, we will avoid the resolution of the fine structure. The other subdomains (extracellular medium, cellular and nuclear membranes, nucleus) have a relatively simple geometry. Even if the size of the cell and the thickness of the cell and nuclear membranes differ by orders of magnitude (the membranes thickness being of the size of the “small” parameter), they can be handled by modern software for solving partial differential equations. So there is no need to include them into the limiting process. The aim of the following considerations is, therefore, to reduce the complex geometry of the cytoplasm while retaining all other aspects of the model in their original form.

For deriving the effective equations in the cytoplasm, we will use techniques motivated by mathematical homogenization of periodic media and Monte-Carlo approaches as used, e.g., in groundwater transport in fractured rocks [11]–[13]. Effectively, this will be done in two steps assuming three well-separated length scales in order to come close to the real geometric structure. Such a strategy is sometimes called *iterative homogenization* [32], [8]. While this idea is not new, to the authors knowledge this is the first time this strategy has been used in a cellular diffusion and reaction model.

The derivation of the effective model of the cytoplasm includes the following steps:

- Find an effective diffusion coefficient, 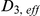, for the averaged cytoplasm.
- Modify the reaction terms and time constant (in such a way that only partial concentrations are taken into account).
- Find the coupling conditions of the averaged cytoplasm to the surrounding membranes.

#### Dimensional Analysis

For a non-dimensionalization, one can use a typical length (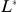), time (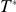), and concentration (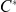). We choose them as follows:

- Length scale: Radius of a cell. It is computed from the volume of a cell under the assumption that the cell has a sperical shape.
- Time scale: We take the diffusion constant in the aqueous part of the cytoplasm as gauge value. This leads to the time scale 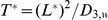.
- Concentration: The choice is not critical since the system is linear in all concentrations. We choose 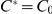.

The mathematical model has formally the same structure as the original with the physical quantities replaces by the scaled parameters. Table 3 provides an overview of the scaled parameters.

**Table 3:** Problem parameters and their scaled values.

parameter	value	scaled
cell radius	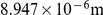	1
membrane thickness	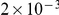	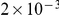
outer radius extracellular medium	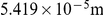	5.0571
volume fraction 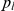	0.254	0.254
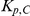	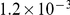	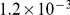
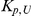	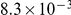	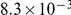
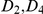	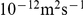	0.004
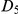	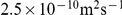	1
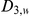	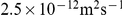	1
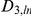	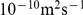	0.4
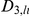	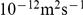	0.004
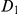	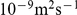	4
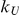	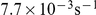	0.0025
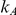	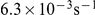	0.002
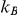	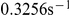	0.1043
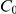	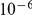	1

#### Reformulation of the System

In the form which the transmission conditions are stated, classical homogenization formulae for periodic structures do not immediately apply. Therefore, we reformulate the system. For the sake of simplicity, we consider only substance 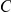 since this system can be solved independently of the others. Once an effective system has been formulated, the same procedure can be repeated with 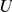 and 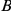, using the results for 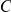.

Let 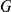 denote the domain under consideration, that is the union of subdomains 1–5. Let 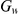 be its aqueous part and 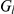 its lipid part. According to our previous notation, 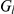 consists of 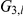 as well as subdomains 2 (cell membrane) and subdomain 4 (nuclear membrane). Similarly, 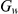 consists of 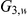 as well as subdomain 1 (extracellular medium) and subdomain 5 (nucleus). On the interfaces between 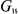 and 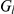, we have transmission conditions of the type
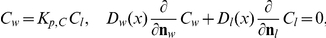
with piecewise constant diffusion coefficients, and 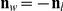. Define now
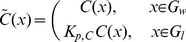
(22)For this new function 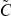, the transmission conditions become
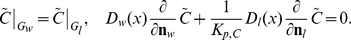
This motivates the definitions
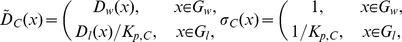


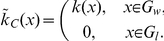
Here, 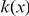 is the collection of all reaction constants. With these definitions, the transmission conditions reduce to continuity of flux and concentration. Hence, the problem can be reformulated as,
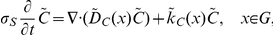
subject to the boundary condition
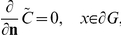
and initial condition
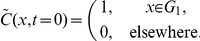



In the following, we will omit the tilda to simplify notation.

For later reference let 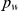 and 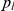 denote the volume fractions of the aqueous and lipid parts, respectively. It holds
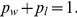
(23)


*Remark.* This reformulation of the transmission conditions and differential equations leads to artificial values for the parameters and concentrations. A direct physical interpretation of these quantities is no longer possible. However, these quantities carry enough information such that certain real values such as compartment contents can be reconstructed. This process will be described later in detail.

#### Averaging on the smallest scale: The First Step

**Figure 2:**
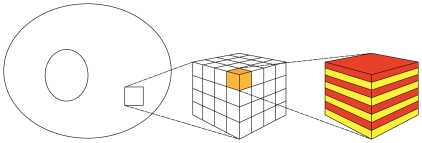
Schematic diagram showing the two step process of iterative homogenization. The first small scale homogenization assumes ideal layered structures representing the membranes (i.e. periodic homogenization; right cube). The second step assumes that these layered structures are tightly packed, with all orientations equally probable, into a model representative subdomain (left cube). A more detailed view is provided in Figure 3 Together these steps allow for an efficient and accurate derivation of effective equations governing the diffusion and reactions in the cytoplasm.

On the smallest scale we assume that the membranes are ideal layered structures as indicated in Figure 2, assumption **A3**. This approximation is motivated by the fact that the organelle membranes create locally densely layered systems throughout the cytoplasm, see for example, [14], [33].

We consider the following situation now: The cytoplasm is assumed to consist of a layered homogeneous structure consisting of lipid and aqueous layers. The thickness of the membranes is considered to be a small parameter 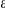. According to the volume fraction, the cytosol layers are assumed to have the thickness 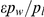. Our aim is to formulate an effective differential equation in subdomain 3 (cytoplasm). Even if the cell and nuclear membranes have the same thickness as the membranes in the cytoplasm, we will not include them in this process. The same holds true for all other parameters.

In [8], a situation of this kind is considered for the stationary problem with boundary conditions including homogeneous Neumann conditions. If we assume that the coordinate system is oriented in such a way that the 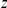-axis is oriented perpendicular to the layers, the limiting equation, for 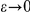, has the form
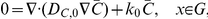
where 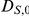 and 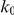 are the coefficients obtained after homogenizing the coefficients individually on all subdomains. On all subdomins, with the exception of 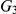, these coefficients are identical to the original ones. On 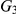, however, standard avareging of stratified media lead to an orthotropic diffusion tensor
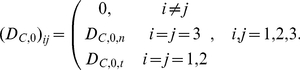
(24)Here,
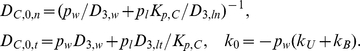
Here, 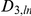 and 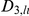 denote the diffusion constants in normal and tangential directions, respectively.

In [9], the parabolic problem without reaction term is considered for the case of “pure periodic” homogenisation and homogeneous Dirichlet boundary conditions. The limiting equation has the form similar to the elliptic case handled above where the coefficient 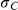 is replaced by its mean value. However, the proof given there can easily be modified to include the present situation.
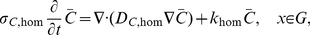
(25)where
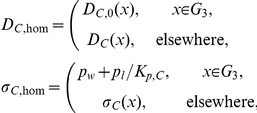


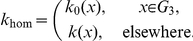



If the orientation of the layers with respect to the coordinate system is different from the one used above, the representation Eq. 24 becomes different. Let 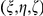 be another cartesian coordinate system. Then there exists an orthogonal matrix 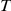 with determinant 1 such that
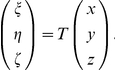
In this new coordinate system, Eq. 25 on 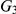 becomes
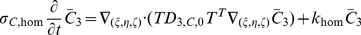
(26)with 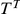 denoting the transpose matrix.

#### Undoing the Reformulation

In order to express the equations again in the untransformed quantities in all of 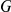 except for the cytoplasm 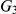, we will undo these transformations. Let
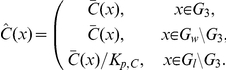
Obviously, on all domains, except for the cytoplasm we obtain 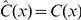 and the original equations from Section 2 back. The transmission conditions for the boundary between extracellular medium and cell membrane as well as between nucleus and nuclear membrane are identical to those of Section 2.

On the boundaries of the cytoplasm, it holds
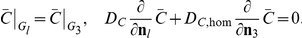
Using the definition of the quantities, these equations are equivalent to, for 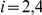,
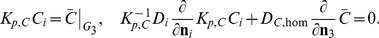
(27)


#### The Second Step

In the previous step, a strict periodic cytoplasm was assumed. This is obviously not true. Instead, at different places in the cytoplasm, the orientation changes. Since we do not have an analytical model, we assume that the orientation is random. In a first approximation we assume further that all orientations are equally probable. The variation in structure of individual cells is considerable. However, the biochemical experiments are carried out using cells in culture corresponding to about 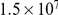 cells per experiment, and the measured data correspond to the joint masses of substances in all cells. This supports the assumption that the orientation of the layered structures at different points in the cytoplasm are independent of each other.

**Figure 3:**
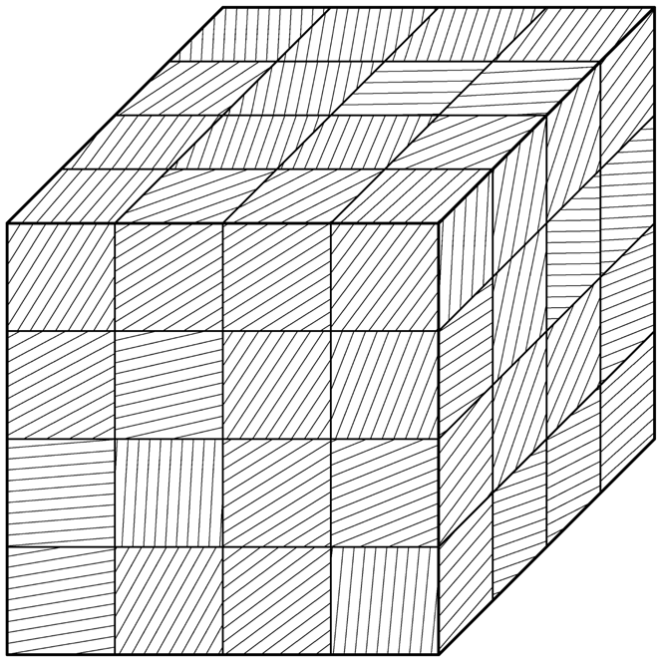
Model domain for random averaging for 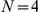
 **.** The orientations of the layers inside the sub-cubes are chosen randomly.

At this point we invoke the next critical assumption **A4**: We assume that the volume is tightly packed with substructures of the type considered before, namely layered materials. The key assumption is that all orientations are equally probable. For the determination of the effective diffusivity, we must use a representative subdomain. It should be small enough to fit into the cytoplasm and being computationally tractable. It must be large enough such that the averaging is justified. Instead of a real 3-dimensional part of the cytoplasm we use a model representative subdomain which is consistent with Assumption **A4**. A part of our model representative subdomain is sketched in Figure 3. We will assume that the substructures are very small compared to the volume of the cytoplasm. Moreover, we will assume that the orientation of the layers is random and uniformly distributed. Since both 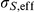 and 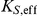 are constant it suffices to consider the stationary problem of determining the effective diffusivity. We will assume that an effective diffusion coefficient exists. In contrast to the first step, no analytic expressions are known in the three-dimensional case. Therefore, the effective diffusion coefficient will be estimated by Monte Carlo techniques.

### Numerical Determination of Effective Diffusivities

Under the assumption that an effective diffusivity for a given problem exists, the corresponding diffusion constants can be determined experimentally. For that, let 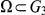 be a subdomain which is in size comparable to the cytoplasm 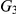 such that the small scale structure is considerably smaller than the size of 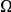. Assume that we want to determine the (scalar) diffusion constant for the diffusion process in 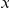-direction. In that case it is convenient to use a cylindrical domain
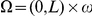
with 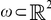 being some bounded domain. On 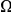 consider the stationary diffusion equation
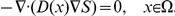



The boundary conditions are selected as follows:

- On the boundary 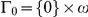, a fixed Dirichlet condition is given,
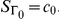

- On the boundary 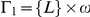, a free diffusion into the surrounding medium is assumed,
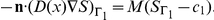
Here, 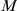 is the mass transfer coefficient and 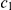 is the concentration in the bulk solution outside of 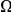.
- All other boundaries 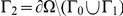 are isolated,
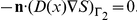
If 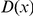 would be a constant 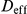, it would hold
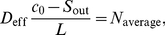
(28)

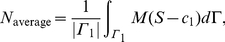
(29)

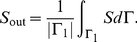
(30)By 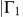 we denote the area of 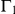. If 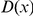 is varying, these equations can be used as an estimation of the effective diffusivity 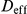. In case of an anisotropic effective diffusivity, the above construction leads to an estimate of the effective diffusivity in 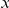-direction, i.e., 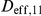.

This numerical procedure has been used in order to obtain an estimation of the error obtained during the first step. Different rectangular parts of real two-dimensional cell microphotographs were used as the computational domain 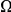 above. The domain was filled with membranes where the geometry was mapped from the photograph. The remaining parts were assumed to be filled by cytosol thus neglecting other components. The effective diffusion constants were estimated according to Eq. 28 and compared to the analytical values according to Eq. 24. The error was in the order of magnitude 5%–20% depending on the folding of the membranes. Details of the experiment can be found in [14].

### The Monte Carlo Experiment

In the cytoplasm we do not have any preferred directions. Therefore, it is convenient to choose a cube as test domain 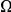 (see Figure 3),
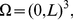
(31)with 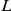 in the order of magnitude of the nucleus diameter. For a given positive integer 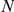, this cube is subdivided into 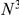 sub-cubes
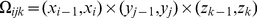
(32)with 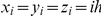 and 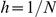. Every subcube is populated with an instance of the homogenized diffusion coefficient 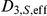 from the first averaging step. According to our assumptions, the orientation of our membranes does not have a preferred direction. Therefore, we will draw rotation matrices 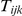 uniformly distributed in the group 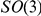 of all rotations such that
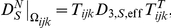
compare Eq. 26. Any rotation in 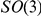 can be described by three angles, the so-called Euler angles. We will use the convention to first rotate around the 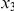-axis by the angle 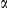, then around the (new) 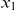-axis by 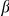, and finally around the new 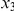-axis by 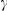. This can be described formally by
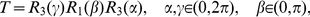
(33)where
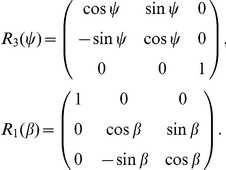
Let 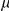 denote the Haar measure on 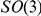 (see [34]). Its density has the simple form
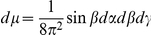
with respect to the Lebesgue measure on 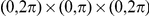. Let 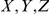 be random variables uniformly distributed on 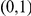. Then, for any realization 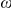, we can set
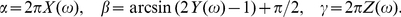
(34)Using the estimation Eq. 28 we can compute the mean value 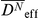. It will hold
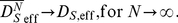
(35)


This algorithm has been tested extensively in [14] in the two-dimensional case. In the two-dimensional case, an analytical solution for the effective diffusion is known [35]. This analytical result has been used as a gauge. The conclusions are:

- The main parameter for the accuracy of the estimation of the effective diffusivity is 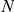. This fact isn't hardly surprising.
- For a given 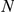, the sample size has only a minor influence on the accuracy. Once a certain number of trials has been reached, the accuracy does not become better. Hence, the optimal sample size seems to be independent of 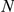.
- The standard deviation for sufficiently large sample sizes roughly halves while doubling 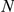. This indicates a linear rate of convergence.
- In all experiments, the mean value of the experimental effective diffusivity is an overestimation of the analytical value.
- If the sample size is too small, the standard deviation is misleadingly small.
- In order to obtain an accuracy of 5% the experiments suggests to use a value of 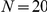 and a sample size of at least 15 trials.
- The estimated effective diffusivity is independent of the choice of 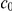, 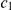, and 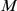.

Finally, we obtain the following equations (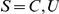) inside of the cytoplasm:
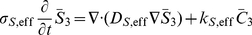
(36)


#### Coupling The Averaged Equations To The Surrounding Subdomains

The transfer conditions are transferred from the periodic homogenization step, namely, Eq. 27. They include continuity of fluxes, the continuity of concentrations taking the partition coefficient into account. The diffusion coefficient for the cytoplasm is taken to be the averaged value after the second step.

### A Compartment Model With Well-Stirred Compartments

Compartment modeling (CM) is a common technique often used to describe transport and reaction in biological systems [36]–[38]. The advantage of using a compartment modelling approach includes decreasing the complexity of the system of equations and thus the computational cost. CM with well-stirred compartments has earlier been used to describe the spatiotemperal dynamics of toxicological or pharmacological active compounds in cells including H
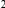
O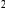 and the anti-cancer agents cisplatin and topotecan [39]–[43]. However, these models have so far only described the diffusion and reaction of relatively water soluble compounds.

In order to validate our PDE model and compare the results with a model based on ordinary differential equations a compartment model describing the above mentioned diffusion and reaction was developed. A well-stirred compartment model is obtained from the detailed model consisting of partial differential equations by using the following assumption:

**A6** The diffusion is very fast compared to the speed of reactions in the system such that the concentration is constant throughout the compartment.

Under Assumption **A6**, the reaction-diffusion equations reduce to simple mass balance equations,
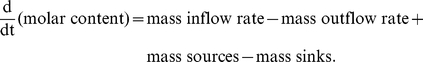



#### Trans-Membrane Diffusion

**Figure 4:**
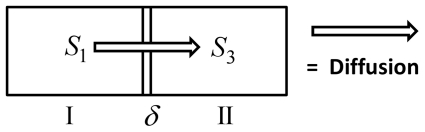
Sketch of membrane diffusion setting. A substance 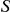 with concentrations 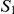 in compartment I and 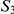 in compartment II is diffusing through a membrane with thickness 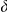.

We consider a thin memfbrane between two compartments as shown in Figure 4. Implementing the use of the partition coefficient 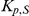, as described earlier we can write the concentration in the membrane at the two boundaries as,
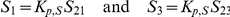
where 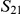 is the concentration at the joint boundary between compartment I and the membrane, where as 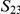 is the concentration at the joint boundary between the membrane and compartment II. If 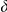 is the thickness of the membrane then the concentration gradient in the membrane will be 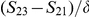. Using Fick's Law of diffusion, which states that the rate at which the material diffuses through any surface is proportional to the product of the area, 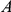, of that surface and the concentration gradient between the two compartments [37], the mass flow rate, 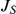, becomes
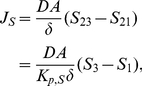
(37)where 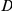 is the diffusion coefficient inside the membrane.

If one of the compartments corresponds to the cytoplasm, say compartment II, the corresponding concentration 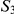 must be taken as the effective concentration 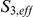 because of the averaging procedure. Note that 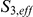 is in general different from 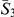 in the averaged equation Eq. 36. As noted earlier, 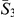 does not have an immediate physical interpretation due to the rescaling but molar contents can be reconstructed from it. Below we will use this reconstructed quantity for defining 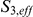.

#### Balance Equations in the Cytoplasm

**Figure 5:**
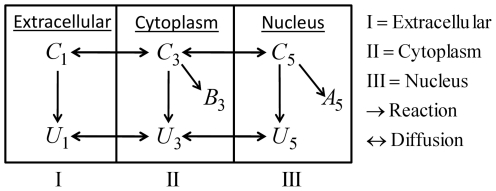
Sketch of compartment system with well-stirred compartments. In the cytoplasm, the effective quantities are used. Cell and nuclear membrane are handled as sketched in Figure 4.

A compartmental system showing the overall dynamics of the system is given in Figure 5. In that figure, we depict the complete reaction and diffusion mechanisms inside and outside of the cell by using a symbolic representation of compartments. All the notations and constants have been taken from the PDE model as shown in Figure 1 and Tables 1 and 2.

Consider first the cytoplasm. We start from the effective reaction-diffusion equation Eq. 36. Integrating over the cytoplasm 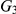 and integrating by parts we obtain
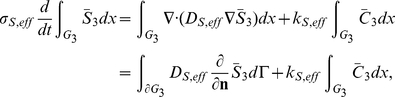
where 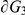 denotes the boundary of 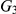. Taking into account the rescaling Eq. 22, we obtain for the molar contents 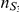 similar as in Eq. 48,
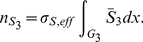
(38)With this definition, by invoking Assumption **A6** an effective concentration can be defined,
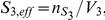
where 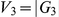 denotes the volume of the cytoplasm. This is the concentration to be used in Eq. 37. With this definition, the mass balance reads
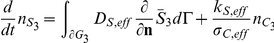
for 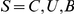. Note that, for 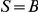, it holds 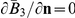 such that the boundary term vanishes. The boundary term in this equation corresponds to mass inflow and outflow while the reaction term corresponds to sources or sinks depending on the sign.

#### Governing Equations

The complete well-stirred compartment model is given by the following equations:

- Compartment I (extracellular medium)
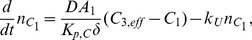
(39)

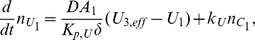
(40)where 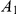 denotes the area of the cell membrane, 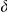 is the thickness of the cell and nucleus membranes, and 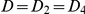 represents the diffusion constant in the membranes. Moreover, 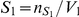 where 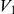 is the volume of compartment I.
- Compartment II (cytoplasm)
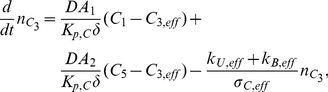
(41)

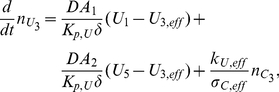
(42)

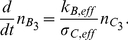
(43)Here, 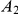 denotes the area of the nuclear membrane. Moreover, 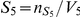 where 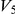 is the volume of compartment III.
- Compartment III (nucleus)
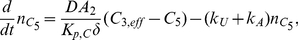
(44)

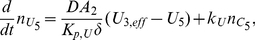
(45)

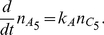
(46)

### The Numerical Realization of The Cell/Environment System

#### The Computational Domain

The mathematical model consisting of the effective partial differential equations has been implemented for the system sketched in Figure 1. We consider one cell together with the surrounding extracellular medium. (cp Table 2).

For the implementation, we used the following assumption:

**A7** The cell is a perfect ball with the different subdomains being spheres.

Furthermore, we assume that the volume of the medium is much larger than the volume of the cells. So we surrounded each cell by an amount of medium which corresponds to volume of medium divided by the number of cells. Furthermore, we assumed that there is no exchange of substance between neighboring cells as well as their surrounding media. This gives rise to no-flux boundary conditions at the outer boundary of the medium. Obviously, the space cannot be filled completely by non-overlapping balls. Here we must assume that the extracellular medium per cell is large compared to the cell such that the exact geometry is unimportant.

Under Assumption **A7**, the three-dimensional problem can be reduced to a one-dimensional computational problem using spherical symmetry. Let us use spherical coordinates with the origin in the center of the cell,
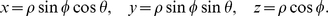
and the diffusion-reaction equations Eq. 23 using the effective diffusion constant from Eq. 35 reduce to
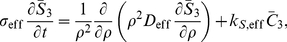
(47)and similarly for the other equations, Eqs. 1–7 in their respective domains.

Because of
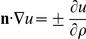
on the surface of a sphere the fluxes can be easily transformed. A description of the computational domain is provided in Tables 4 and 2. The boundary conditions for the substances 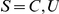 are summarized in Table 5. Boundary conditions for 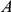 are handled accordingly.

**Table 4:** Definition of the computational domain.

constant	value	comments
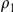 [m]	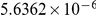	Radius of a ball with volume of nucleus, Table 2
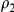 [m]	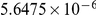	
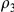 [m]	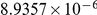	
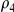 [m]	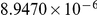	Radius of a ball with volume of cell, Table 2
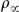 [m]	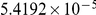	

The missing values have been computed as follows. The thickness of the membranes, 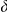, has been determined by multiplying 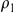 by the relative thickness of the nuclear membrane from Table 2. Then, 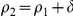 and 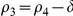. For 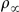, the amount of cell medium per cell has been computed. 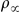 is the radius of a ball with that volume.

The nucleaus is described by 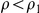, the nuclear membrane by 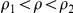, the cytoplasm by 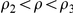, the cell menbrane by 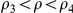, and the extracellular medium by 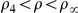.

**Table 5:** Summary of boundary conditions.

boundary	boundary/transmission conditions
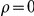	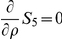
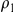	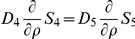
	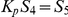
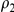	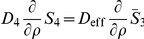
	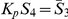
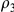	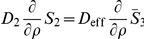
	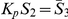
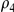	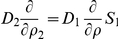
	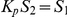
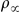	physical conditions

#### Computing The Molar Content

Instead of the concentrations 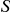, the molar content 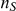 of the individual species is measured in the experiments. For a given subdomain 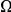, the molar content is given by
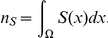
Since we are using spherical symmetry, the integral over a sperical domain 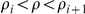 reduces to
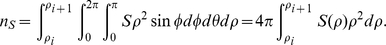
In the cytoplasm, we must take into account the averaging together with the rescaling Eq. 22 such that, for the effective quantities, the molar content becomes
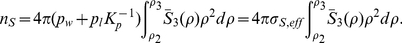
(48)For the evaluation of this integral, Comsol Multiphysics provides standardroutines.

#### Physical Boundary Conditions And Initial Values

The direct translation of the conditions of Section Boundary and Initial Conditions becomes:

- boundary conditions:
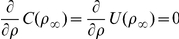

- initial conditions:
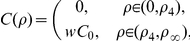


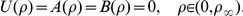

The weight function 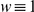 corresponds to Eq. 21. This function 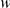 can also be chosen to be
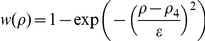
where 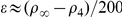. While not changing the initial value much the latter choice speeds up the computations in Comsol Multiphysics a lot.

For 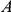, Eq. 16 becomes
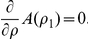



#### Realization In Comsol Multiphysics

The model was implemented in Comsol Multiphysics 3.5 [44] using the scripting language and the Reaction Engineering Laboratory. This software uses the finite element method for discretizing with respect to the spacial independent variable. The time stepper is DASSL which implements a variable order, variable step method [45]. Using the reduction to one dimension, the singularity of Eq. 47 near 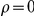 can be avoided by multiplying through by 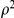.

A more severe problem is the introduction of the partition coefficients in the boundary values according to Table 5. Here, we use a proposal from Comsol Multiphysics' model library [46]. Take as an example the boundary conditions near 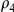,
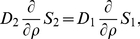
(49)

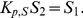
(50)For a given (large) constant 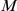, these boundary conditions will be replaced by
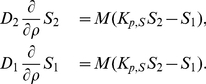
(51)Eq. 51 ensures continuity of mass flow such that the conservation of mass is secured. However, Eq. 50 is only approximately satisfied. The larger 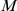, the better the approximation. This penalty approach is easily implementable in Comsol Multiphysics.

Comsol Multiphysics uses the method of lines with the finite element method for the spatial discretization of the differential equations. In the numerical experiments, we used second order Lagrange elements. The cell and the nuclear membrane were discretized using 20 elements on each while, in the other subdomains, 100 elements have been used. This leads to a system of ordinary differential equations with 6188 equations.

The realization of only partially defined variables in Comsol Multiphysics is very convenient by using the possibility of restricting the validity of some variables to certain subdomains. For example, 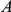 is only defined inside of the nucleus and nowhere else. Thus it will not appear as a dependent variable in the other subdomains.

The complete code is available on request.

#### Realization of The Well-Stirred Model

A compartment model is arguably the most efficient computational treatment of a cell. In order to compare the PDE and compartment model, the latter Eqs. 39–46 have been implemented and numerically solved using Matlab [47]. As with the PDE model, all the chemical and physical constants have been taken from Tables 1 and 2, respectively.

## Results And Discussion

### Effective Equations

The geometry of the intracellular compartments is very complex and a full model including all the PDEs for describing the diffusion and reactions throughout the cytoplasm is practically impossible. This problem can be resolved by using the techniques described above resulting in a simplified geometry and effective diffusion and reaction equations. This approach has been shown to reduce the computation time more than 5000-fold compared to a fully detailed model in modelling spatio-temporal signalling in rod phototransduction [48]. The present approach makes it feasible to solve much more complex models on much more complex domains.

To estimate the effective diffusion coefficient numerical experiments with real membrane structures taken from electron microscopy photographs were previously performed [14]. They show that the first averaging step from the finest to the intermediate level introduces an error in the diffusion constant in the order of magnitude 5%–20% depending on the folding of the membranes. Therefore, we are interested in an approximation of the effective diffusivity with accuracy in the same order of magnitude. The estimation of effective diffusion coefficients in the cytoplasm is done in a preprocessing step using the above introduced Monte Carlo procedure. The resulting effective diffusion constants are
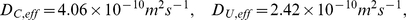


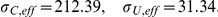
Even if the physical diffusivities of 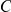 and 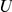 are taken to be equal, the effective diffusivities differ because of the different partition coefficients. It is important to note that these constants do not have any real physical significance because they are based on the rescaling discussed previously when simplifying the transmission conditions. Their importance lies in the fact that, by using these values in the averaged equations, the real averaged concentrations inside the cytoplasm can be reconstructed.

In order to successfully apply the our averaging method a number of assumptions were taken and their justification warrants some discussion. The first two assumptions are related to the intracellular geometry, more specifically the organization and distribution of membranes and organelles and their relation. Looking at the pictures in [14], [33] the approximation of periodically layered membranes can be justified if we also in the term cytoplasm include the interior of the organelles. Further the same picture gives support to an equal probability of all orientations of these sub-structures in a larger scale. The difference in scale between these substructures and the volume of the cytoplasm is justified by the fact that the thickness of a membrane is in the order of a few nm while the dimension of a typical organelle such as mitochondria is measured in 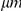.

The third assumption concerns the homogeneity of the biophysical properties of the cytoplasm and the membranes. This is a simplification given that compartmentalization exists in all cellular subdomains [49], [50]. The impact of compartmentalized reaction and metabolism both in the cytoplasm and membrane poses a significant challenge both mathematically and computationally.

The two remaining critical assumptions are related to how the molecular interactions and reactions are modeled. Based on measurements from cellular experiments the initial number of molecules per cell of PAH DE (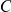) and the over expressed GST enzyme are about 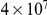 and 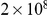, respectively. To account for the interactions and reactions of all these molecules as individual molecular collisions would become very computationally costly if not practically impossible. Metabolic processes involving large numbers of molecules are successfully modeled deterministically using concentration as a descriptor. In addition, the numbers shown above are close the range of the suggested 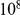
–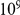 molecules that may be accurately modeled using concentrations [51]. However, Gillespie [52] emphasizes that the question if the deterministic model can be used instead of a stochastic description can, up to now, only be decided if both models are solved and the trajectories generated by the deterministic model are approximating the stochastic ones sufficiently accurate. The uses of a partition coefficient to describe the behavior at the interface between the two compartments is based on the idea that the processes of absorption and desorption of the individual species into or out of the membrane are in rapid equilibrium.

### Simulation Results

**Figure 6:**
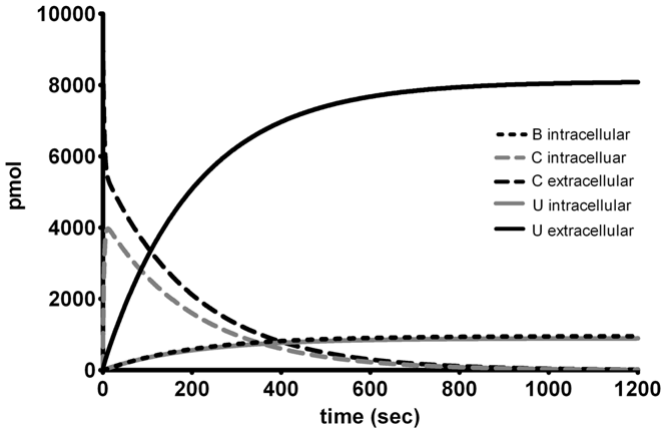
Extra and intra cellular profiles of BPDE and its metabolites obtained from the PDE model. The model was run using constants and expressions as described in Tables 1 and 2 and the different species subsequently plotted.

In order to set the model to mimic the cellular exposure, uptake, metabolism and reaction of the prototype PAH DE, benzo[a]pyrene diol epoxide (BPDE), data from *in vitro* experiments and cells in culture describing the partitioning, intracellular metabolism, and reactivity of BPDE were collected. The constants used can be found in Tables 1 and 2. The results from the PDE model show a rapid uptake and reaction of BPDE (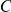) (Figure 6). The rapid uptake results in an intracellular profile of BPDE showing maximum levels reached within 1 min followed by a slower decrease. Concurrent with the full depletion of both extra and intra cellular BPDE, maximum levels of GSH conjugates (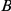) and tetrols (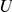) are reached after 10 min. As can be seen the major compartment of reaction is the extracellular medium where about 70% of the added BPDE endup as tetrols (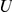). Comparing the amounts of tetrols formed extra cellular to intra cellular shows almost 6 times more formed in the former. This is in agreement with the more than 200-fold larger extracellular volume compared to the intracellular. Furthermore, the more hydrophilic properties of the tetrols (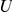) favor an extracellular distribution. Although the fact that most of the hydrolysis of BPDE occurs extracellularly might seem trivial this is not always appreciated when interpreting in vitro experiments.

### The PDE model vs Well-Stirred Compartments

**Figure 7:**
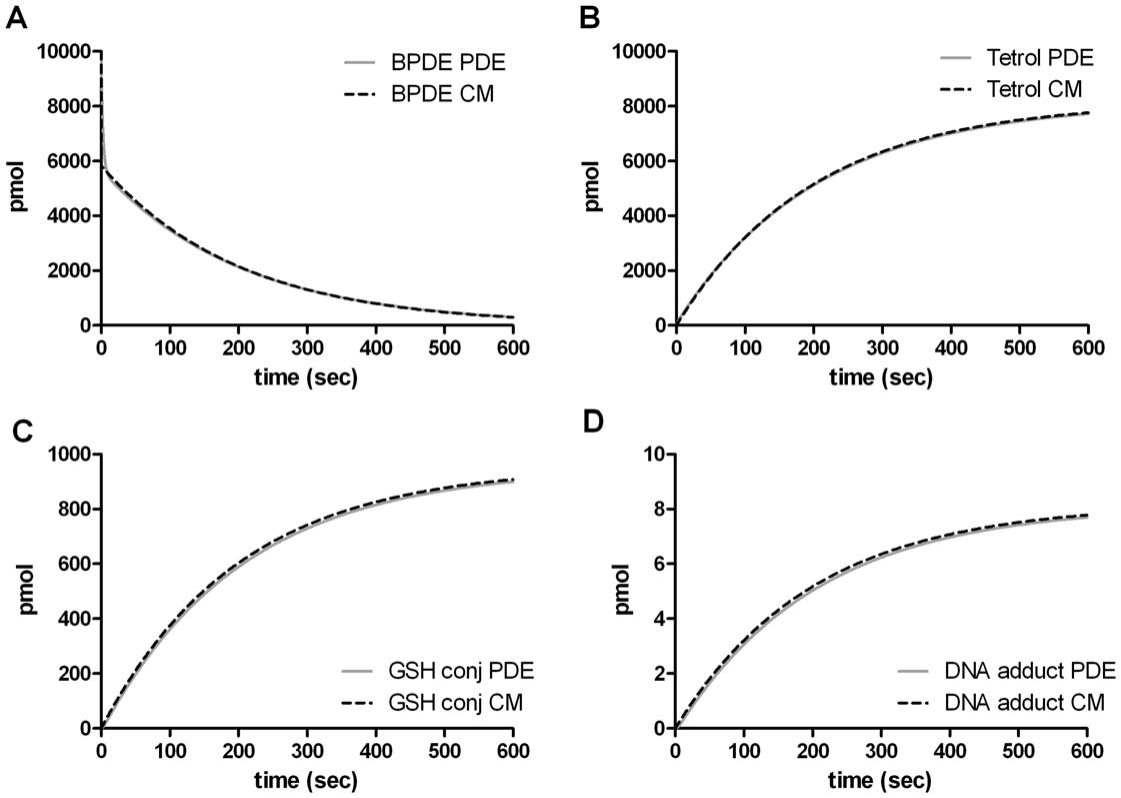
Comparison between the compartment model and the PDE model. The parameters are taken from Tables 1 and 2. The individual panels show A the degradation of BPDE in extracellular compartment, B formation of tetrols in extracellular compartment, C formation of glutathione conjugates in cytoplasm, and D formation of DNA adducts in the nucleus.

**Figure 8:**
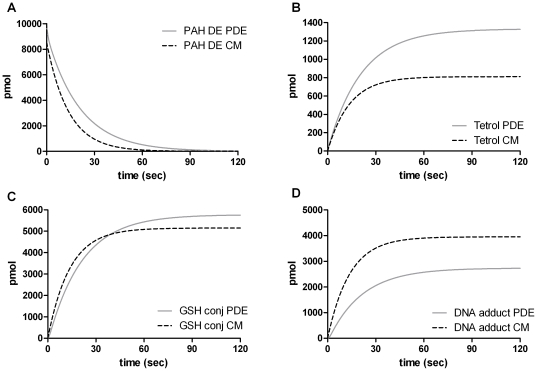
Comparison between the compartment model and the PDE model. The parameters were changed according to Table 6 to describe enhanced diffusion and reactivity of PAH DE (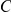). The panels are ordered in the same way as in Figure 7.

The Eqs. 39–46 have been implemented in Matlab. As with the PDE model, all the chemical and physical constants have been taken from Tables 1 and 2, respectively. Comparing the numerical results with the PDE model shows a nice agreement between the two models (Figure 7) at certain parameter values. However, when examining parameters describing enhanced diffusion and reactivity of 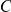, (shown in Table 6), the two models displayed differences (as can be seen in Figure 8). For example, the compartment model displays a faster uptake of 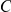 leading to lower levels of extracellular tetrols (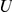) (Figure 8 (a) and (b)). In a similar fashion the transport of 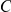 between the cytoplasm and nucleus is faster thus reducing the levels of GSH conjugates (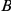) and increasing the levels of DNA adducts (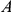) (Figure 8 (c) and (d)).

**Table 6:** Modified parameters for the compartment model.

constant	value
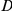 [m 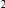 s 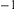 ]	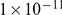
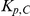	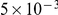
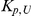	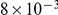
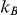 [M 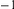 s 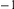 ]	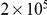
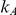 [s 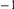 ]	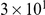

**Figure 9:**
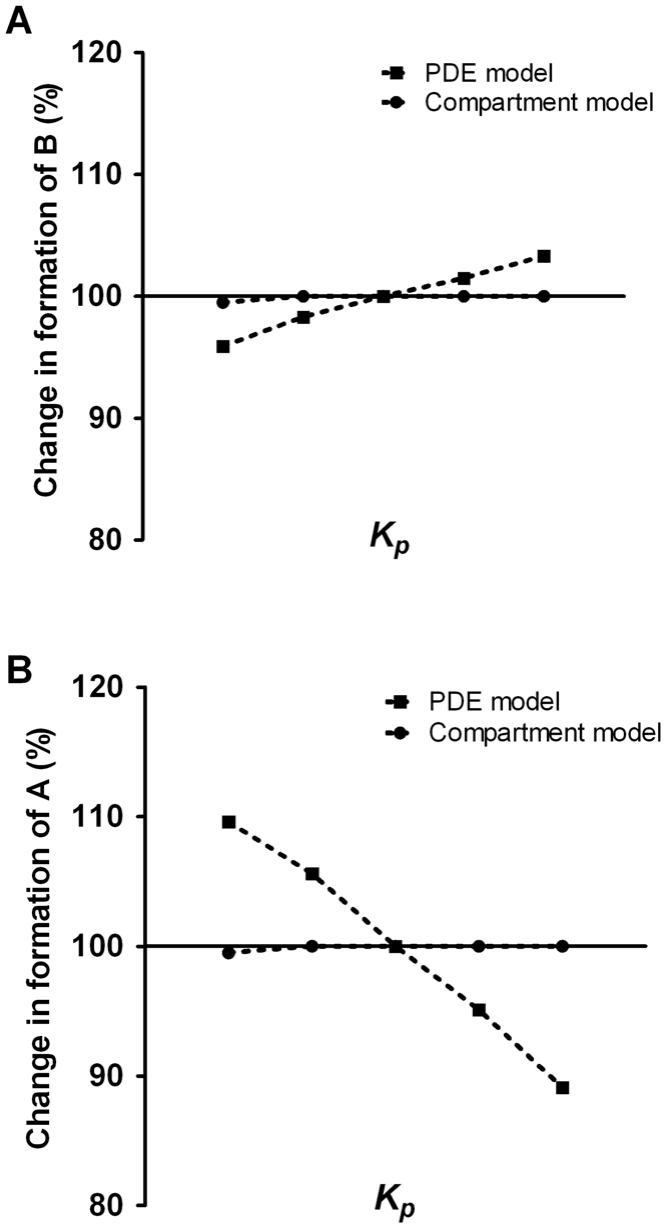
Comparison between the compartment model and the PDE model. Effects on the formation of glutathione conjugates (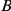) and DNA adducts (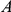) from increasing partition coefficient (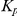) of PAH DE (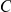) are shown in A and B, respectively. The values for 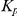 are taken from Table 7. The other parameters are fixed according to Table 6.

The inability of the CM to respond to certain parameter values regarding the intracellular dynamics of these lipophilic compounds was further shown when comparing the effect of increasing 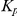. The PDE and CM were run using the parameters in Tables 2 and 6 with 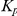 changed according to Table 7. As can be seen in Figure 9, using the compartment model, neither the formation of 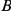 or 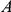 was affected by changes in lipophilicity while the PDE behaved as outlined. This lack of response to changes can be explained by the fact that the membranes have no apparent role in the compartment model. The basic assumption for the well-stirred compartment model is that the diffusion process is much faster than the reactions involved. The above results indicate that this assumption is not justified for certain values of the parameters such that this well-stirred model no longer describes the metabolism/reactions correctly. Although it remains to be verified experimentally, the PDE model thus has the potential to describe the dynamics of very reactive and hydrophobic carinogenic polycyclic aromatic hydrocarbon diol epoxides.

**Table 7:** Parameters varied.

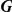 [M]	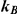 [ 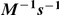 ]	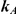 [ 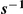 ]	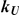 [ 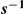 ]	% 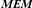	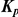
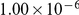	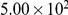	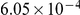	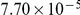	10.0	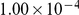
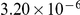	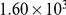	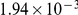	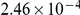	15.0	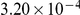
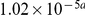	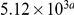	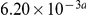	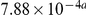	22.4^a^	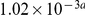
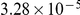	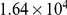	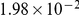	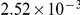	33.4	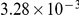
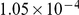	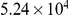	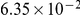	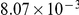	50.0	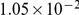

a used as baseline values.

Parameters varied to study the effect on GSH conjugation and DNA-adduct formation.

### Parameter dependence

**Figure 10:**
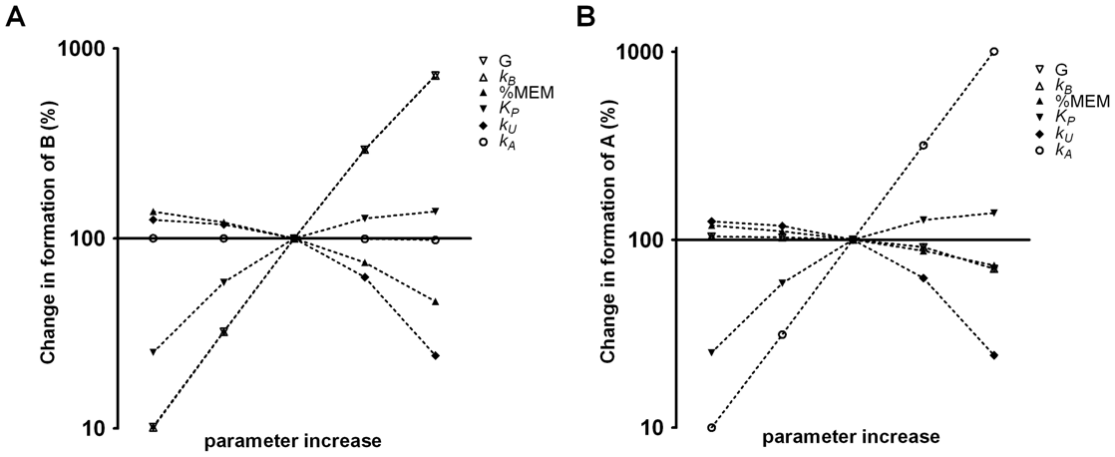
Change in formation of 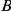
 **(A) and** 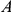 **(B) resulting from change in parameter value.** The PDE model was run using constants as described in Table 7.

To test the responsiveness of the system selected input parameters of experimental interest, found in Tables 1 and 2, were varied. The range of variation for the separate parameters was chosen from available data representing different PAH DEs and representative cell conditions [18]–[21]. To maintain realistic values of the parameters tested %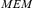 was varied between 10% and 50% while the rest were varied about 100-fold (from the highest and lowest values, Table 6). For example, in the case of the solvolytic reactivity, 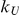, the highest value (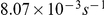) represents the reactivity of BPDE and the lowest value (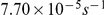) the reactivity of the much less reactive DEs from benzo[c]phenanthrene (BPhDE). The parameters describing the level of GST enzyme expressed and percentage of cellular membrane, 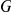 and %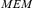, are cell specific and thus represents the cellular heterogeneity. 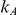, 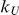 and 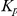 are PAH DE specific and represents different scenarios of exposure. 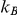 depends on the catalytic efficiency of the enzyme expressed in the cell towards the compound used. Since the parameters %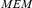 and 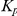 both affect the distribution of the modeled compounds (between membrane and cytoplasm) it also affects 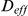. Accordingly, specific values of 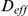 were calculated in each case. The values of the parameters were varied one at a time while keeping the remaining parameters fixed at baseline values. To consider the impact on the different compartments the enzymatic detoxification reaction by GST in the cytoplasm (formation of 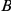) and the reaction with DNA in the nucleus (formation of 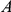) were modeled (Figure 10).

As can be seen for GSH conjugate formation (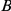) (Figure 10 (a)) it is directly proportional to the enzymatic reaction rate constant and amount of enzyme governing its formation as expected. The major competing pathway of hydrolysis becomes incrementally more effective for more easily hydrolysable substances (increased 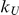) whereas the reaction with DNA (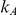), representing a minor pathway, does not affect conjugation. Partitioning into the membrane (as represented by a lower 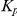) and an increased membrane fraction leads to lower conjugate formation as metabolism does not occur in the membrane. As a consequence conjugation occurs over longer time periods. The influence of these parameters on conjugate formation are thus consistent with what can be expected but reveal that the influences are quantitatively different. The rank order of importance for conjugation are high enzyme and catalytic efficiency, slow hydrolysis, high water solubility, low cell membrane content, whereas DNA binding is of no significance.

**Figure 11:**
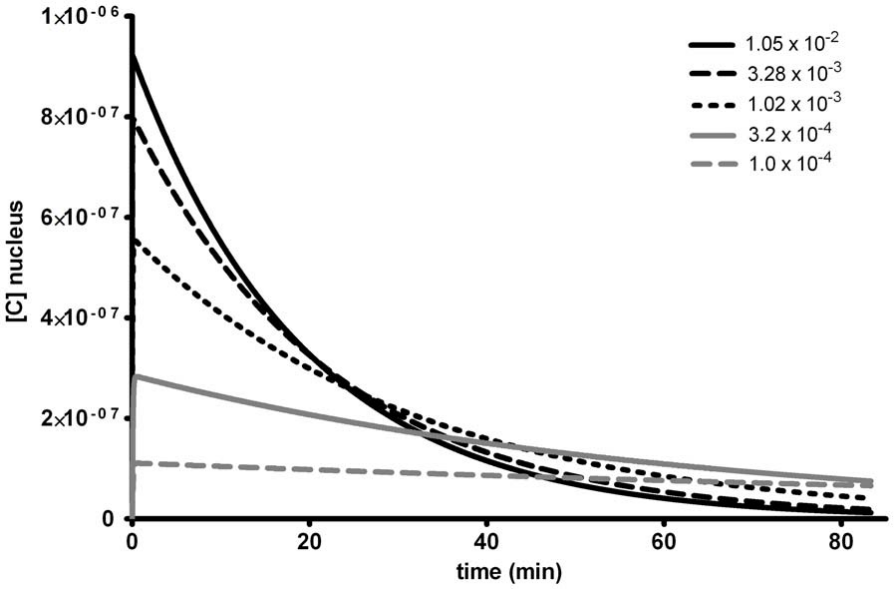
Effect of increased lipophilicity ( 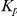
 **) on nuclear concentration profile of PAH DE (** 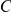 **).** The values for 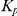 are taken from Table 6, the other parameters are fixed according to Tables 1 and 2.

The same analysis of DNA binding (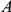) similarly shows that the chemical reaction rate constant (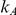) is most important (Figure 10(b)). To prevent DNA modification the hydrolysis rate (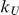) contributes more than enzyme efficiency (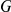, 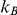). A high membrane fraction prevents DNA binding and higher water solubility promotes DNA binding as expected but the influences are marginal. The most striking finding from this analysis is that the hydrolysis and conjugation efficiency appear to result in semi-treshold effects on DNA binding. That is, only the highest values start to affect DNA binding efficiently. It is conceivable that lipid partitioning allows a protected transport pathway that, although lowering availability for both conjugation/hydrolysis and DNA binding, favours the latter at the expense of detoxication. Indeed, modelling the effect of increased lipophilicity on the nuclear concentration of PAH DE (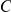) further supports this notion (Figure 11). Already after about 1 h the concentration of the more lipohilic compounds are higher in the nucleus compared to those that are more water soluble. For the glutathione transferases (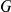) a possible location in the nucleus (which has been suggested [53]) might thus be of particular significance for efficient cancer protection.

In summary, the analysis of parameter sensitivity supports the validity of our PDE model and suggests new ideas on cellular processes governing the dynamics of lipophilic compounds. These ideas of course have to be tested by experiments.

### Comparison To Results From Cellular Experiments

**Figure 12:**
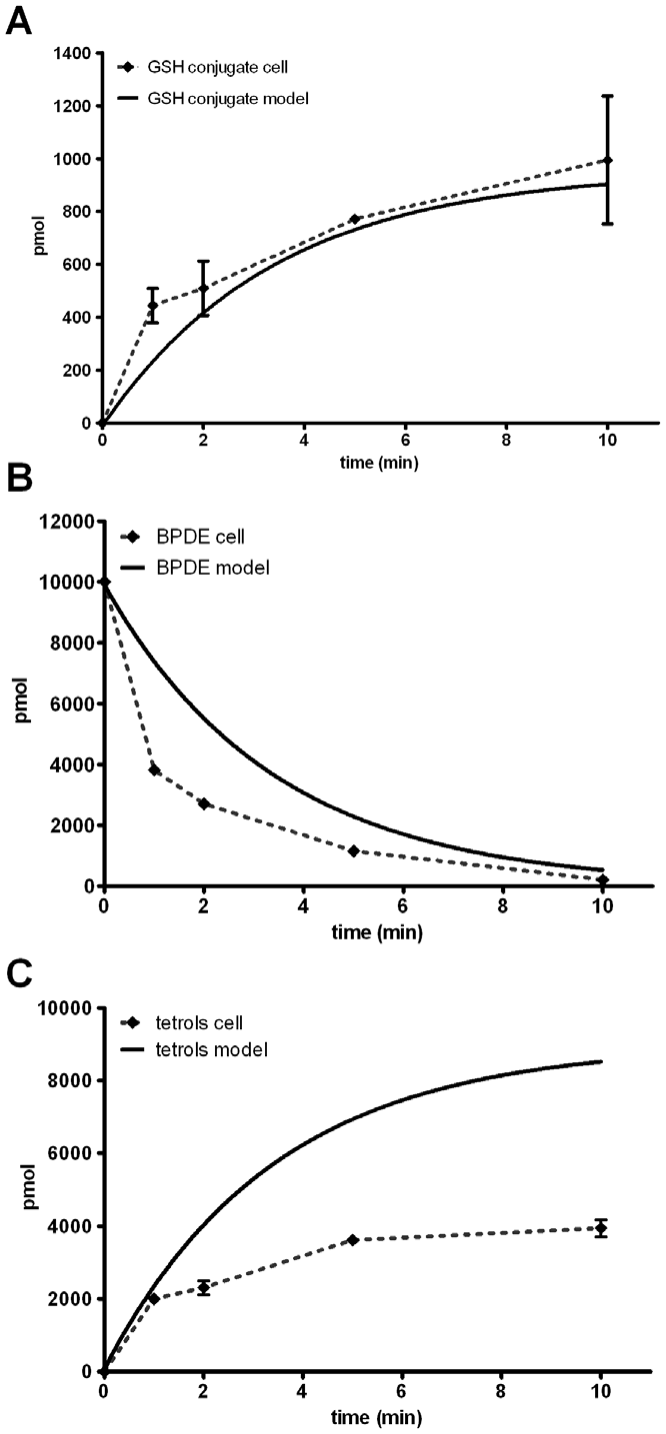
Comparison between results from the PDE model and cells. Simulated amounts of the different species were generated by running the model for 600 s using constants and expressions in Tables 1 and 2 and subsequently plotted. Results from cellular experiments show mean 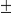 SEM, 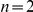.

Comparisons between the results from the PDE model with the actual results from our experiments using mammalian cells are shown in Figure 12. The results from the cellular experiments were in part published previously [20]. In short, mammalian V79 cells stably expressing the human GST P1-1 were exposed to 1 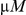 (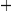)-*anti*-BPDE. At different time points cells and medium was harvested in order to analyze the metabolism and reaction of the diol epoxides. When measurements of the different species were performed the total amounts of BPDE, tetrols and GSH conjugates were analyzed. Accordingly, the model was made to mimic this situation by adding the intra and extra cellular profiles.

Comparing the formation of GSH conjugates, the model demonstrates reasonable agreement with the results of the cellular measurements, both showing a rapid initial formation of conjugates reaching about 1000 pmol after 10 minutes (Figure 12 (a)). Moreover, the conversion of BPDE shows the same rapid progression in both model and in cells (Figure 12 (b)). The only major difference between the model and the cells is the formation of tetrols. Although in qualitative agreement, the model shows a 2-fold higher amount of tetrols formed (Figure 12 (c)). This can in part be explained by the fact that BPDE also reacts with other cellular macromolecules, such as proteins. Earlier studies performed in different cell-like systems have shown that up to about 10% of the total reaction of BPDE can be accounted for as covalent binding to proteins [54], [55]. In addition, the recovery of the measured metabolites from the cellular experiments was usually around 80%, together explaining the majority of the discrepancy. These initial observations suggest that the model reasonably predicts the behaviour of a reactive molecule added to a cellular system. Clearly these predictions need to, and will be improved, as the model is refined and experimental issues addressed. These include a complete set of data for the fate of BPDE and its reaction products in engineered cellular systems.

### Conclusions

Here we present a mathematical model describing the diffusion and reactions of toxic and lipophilic compounds in an effort to identify parameters determining biotransformation and toxicity of such compounds. To our knowledge this is the first model including the cytoplasmic membranes in a diffusion reaction model and thus making it possible to study the effect of partitioning. In order to make the system numerically treatable, techniques motivated by mathematical homogenization were applied and an effective diffusion coefficient was estimated. This reduction in complexity allowed for an easy treatment of the resulting equations with standard tools for the numerical solution of partial differential equations. The use of more general cell shapes than balls does not pose any new difficulties. Furthermore a corresponding well-stirred compartment model was not able to respond to parameters governing the intracellular dynamics of lipophilic compunds further strengthening the need of the developed PDE model.

In [10], a periodic homogenization problem for the cytoplasm has been considered. The mathematical model for the dynamics of intracellular calcium concentration considered there leads to a set of equations for the concentrations of calcium ions in the cytoplasm and the endoplasmic reticulum together with transmission between these subdomains which is similar to the mathematical description in our model. In the homogenized limit, the effective equations become the bidomain equations. In the present paper, the transmission conditions are of a simpler form. Therefore, we use a different approach which leads to a single diffusion-reaction equation for each species.

As a model compound, we used benzo[*a*]pyrene diol epoxide (BPDE) a prototype for studying the different toxic and carcinogenic effects of PAHs. The general applicability of the model and the mathematical approach was validated by comparing the *in silico* results to results from experiments performed in mammalian cells. Lipophilicity was identified as an important parameter in both metabolism and formation of DNA adducts. In general the numerical results show good qualitative and quantitative agreement with the cellular measurements.

Ultimately, with a set of established parameters describing physicochemical and metabolic preferences this model can describe the diffusion and reaction of any lipophilic and potentially toxic compound. In addition, the model can help in determining detailed kinetic parameters difficult to obtain experimentally. In this article we have focused on establishing a mathematical model to study the effect of partitioning on reactions and metabolism of the ultimate carcinogenic metabolite BPDE. In the future, we are planning to implement a higher level of complexity by including protein binding, enzymatic compartmentalization and modeling the diffusion and reactions of the parent PAHs. Furthermore, the model will be trained to describe DNA repair on available data. This can easily be accomplished by including more reactants, and more complex reaction chains including membrane surface coupled biotransformation. Finally, our approach is hoped to yield a modelling environment that, verified against a set of well defined chemical and enzymatic processes occuring in metabolically engineered mammalian cells with defined compartmentation, will be generally applicable.
